# Enhanced field electron emission properties of hierarchically structured MWCNT-based cold cathodes

**DOI:** 10.1186/1556-276X-9-55

**Published:** 2014-02-01

**Authors:** Loïck-Alexandre Gautier, Vincent Le Borgne, Samir Al Moussalami, My Ali El Khakani

**Affiliations:** 1 Institut National de la Recherche Scientifique, Centre Énergie, Matériaux et Télécommunications, 1650 Blvd. Lionel–Boulet, Varennes, Quebec J3X-1S2, Canada; 2 pDevices Inc, 75 Blvd. de Mortagne, Suite 108, Boucherville, Quebec J4B 6Y4, Canada

**Keywords:** Vertically aligned carbon nanotubes, Plasma enhanced vacuum deposition, Hierarchical structuring, Field electron emission, Cold cathode

## Abstract

Hierarchically structured MWCNT (h-MWCNT)-based cold cathodes were successfully achieved by means of a relatively simple and highly effective approach consisting of the appropriate combination of KOH-based pyramidal texturing of Si (100) substrates and PECVD growth of vertically aligned MWCNTs. By controlling the aspect ratio (AR) of the Si pyramids, we were able to tune the field electron emission (FEE) properties of the h-MWCNT cathodes. Indeed, when the AR is increased from 0 (flat Si) to 0.6, not only the emitted current density was found to increase exponentially, but more importantly its associated threshold field (TF) was reduced from 3.52 V/μm to reach a value as low as 1.95 V/μm. The analysis of the *J*-*E* emission curves in the light of the conventional Fowler-Nordheim model revealed the existence of two distinct low-field (LF) and high-field (HF) FEE regimes. In both regimes, the hierarchical structuring was found to increase significantly the associated *β*_LF_ and *β*_HF_ field enhancement factors of the h-MWCNT cathodes (by a factor of 1.7 and 2.2, respectively). Pyramidal texturing of the cathodes is believed to favor vacuum space charge effects, which could be invoked to account for the significant enhancement of the FEE, particularly in the HF regime where a *β*_HF_ as high as 6,980 was obtained for the highest AR value of 0.6.

## Background

Carbon nanotubes (CNTs) are known to exhibit a unique combination of properties that make them a material of choice for field electron emission (FEE) applications. Indeed, their low *Z* atomic number, unequalled aspect ratio (of up to?≥?10^4^), and high charge carrier mobility along with their mechanical strength and stiffness are highly attractive for a variety of applications, such as cold cathode emitters for lighting devices (Cho et al. [1]; Bonard et al. [2]; Saito & Uemura [3]), field emission displays (Lee et al. [4]; Choi et al. [5]) and miniature X-ray sources (Jeong et al. [6]; Sugie et al. [7]; Yue et al. [8]). When used as electron emitters, multi-wall carbon nanotubes (MWCNTs) are preferred to single-wall carbon nanotubes (SWCNTs), because of their metallic-like behavior and their multi-layered structure, which confers them higher resistance to degradation (by at least a factor of 10) (Bonard et al. [9]). In order to further enhance the FEE performance of MWCNTs, strategies are being developed to either increase their electron current density or, even better, reduce their associated threshold field (TF). In this context, researchers have proposed different approaches, including strategies to increase the aspect ratio of the nanotubes (Jo et al. [10]), to chemically functionalize them (Jha et al. [11]) or to tailor their growth sites through patterning techniques (Hazra et al. [12]). In particular, to reduce the threshold field and thereby the power consumption of the FEE devices, microfabrication techniques were often used and shown to be effective in reaching reasonably low TF values (in the 2 to 3 V/μm range) (Zhang et al. [13]; Sanborn et al. [14]; Choi et al. [5]). Such microfabrication-based approaches, though they enable precise microtailoring of the shape of emitting tips, are costly and involve relatively complex multi-step plasma processing. Previous studies have shown that the TF of CNTs is affected by the shape of the emitters (Chen et al. [15]; Futaba et al. [16]) and their surface density through the screening effect (Hazra et al. [12]; Pandey et al. [17]). By tailoring the emission sites as well as changing their density, it is possible to minimize this screening effect that can adversely affect the FEE properties of the CNT samples (Bonard et al. [18]).

In the present paper, we report on a relatively simple, fast, efficient, and very cost-effective approach to achieve CNT-based cold cathodes exhibiting very low threshold fields. Our approach is based on a hierarchical structuring of the emitting cathode, which consists of a pyramidal texturing of a silicon surface by optimized KOH chemical etching followed by a *plasma-enhanced chemical vapor deposition* (PECVD) growth of MWCNTs on the Si pyramids. This approach offers the advantage of not only increasing the aspect ratio of the emitting structures, but also increasing significantly the effective electron emitting surface. By investigating the FEE of these novel hierarchal MWCNT (h-MWCNT) cathodes, in particular as a function of the initial aspect ratio of the Si pyramids, we were able to optimize their TF and reach a value as low as 1.95 V/μm, with a very easily affordable process.

## Methods

### Fabrication of hierarchically structured MWCNT-based cold cathodes

To fabricate the h-MWCNT cathodes, we have first performed a KOH etching (under optimized conditions of 30-min etching time at 90°C in a 8 wt.% KOH solution) of mirror-polished and n-doped Si (100) wafers (0.001 to 0.005 Ω·cm) to transform their initial smooth surface into pyramids (with heights of several micrometers), randomly and homogeneously distributed over all the treated Si surface. To control the pyramid aspect ratio (AR, defined as the ratio of their height to their base-width), the KOH-etched Si substrates were subjected to precise mechanical polishing. Thus, the Si substrates with various AR values (ranging from sharp pyramids to flat-topped ones (mesas)) were obtained. Prior to the PECVD growth of the MWCNTs, 3D-textured Si substrates were catalyzed by coating them first with a sputter-deposited thin Al film (20 nm) and by post-annealing them at 500°C for 30 min under air. Then, an Fe-catalyst nanoparticle film (with a nominal thickness of approximately 25 nm) was deposited by means of pulsed laser deposition (Dolbec et al. [19]; Aïssa et al. [20]). These Fe/Al_*x*_O_*y*_/Si-catalyzed substrates were introduced into a PECVD reactor, operating at 13.56 MHz, for CNT growth under the following operating conditions: substrate temperature of 700°C, gas flow of 500 sccm (Ar)/20 sccm (H_2_)/5 sccm (C_2_H_2_) at a total pressure of 600 mTorr, an applied RF power density of 0.44 W/cm^2^, and a substrate biasing of −40 V. These conditions were found to lead to the growth of vertically aligned MWCNTs onto flat Si substrates with a length of approximately 2.8 μm.

### Characterization of the FEE properties of the h-MWCNT cold cathodes

The FEE properties of the MWCNTs grown on both pyramidally textured (with various AR values) and flat silicon (used as a reference sample having AR value of zero) substrates were systematically characterized in our FEE measurement setup, which is equipped with a high-precision translation stage that positions the MWCNT emitters at 100 ± 0.4 μm from the upper copper collecting electrode. The FEE measurement chamber was pumped down to 5.10^−6^-Torr base pressure before proceeding with the measurements. An increasing voltage was then applied from 0 up to 400 V, and all the samples were cycled several times until a stable FEE regime is reached to allow meaningful comparison between the samples. This cycling of the MWCNT cathodes enables soft and progressive cleaning of the MWCNTs (Collazo et al. [21]).

## Results and discussion

**Figure 1:**
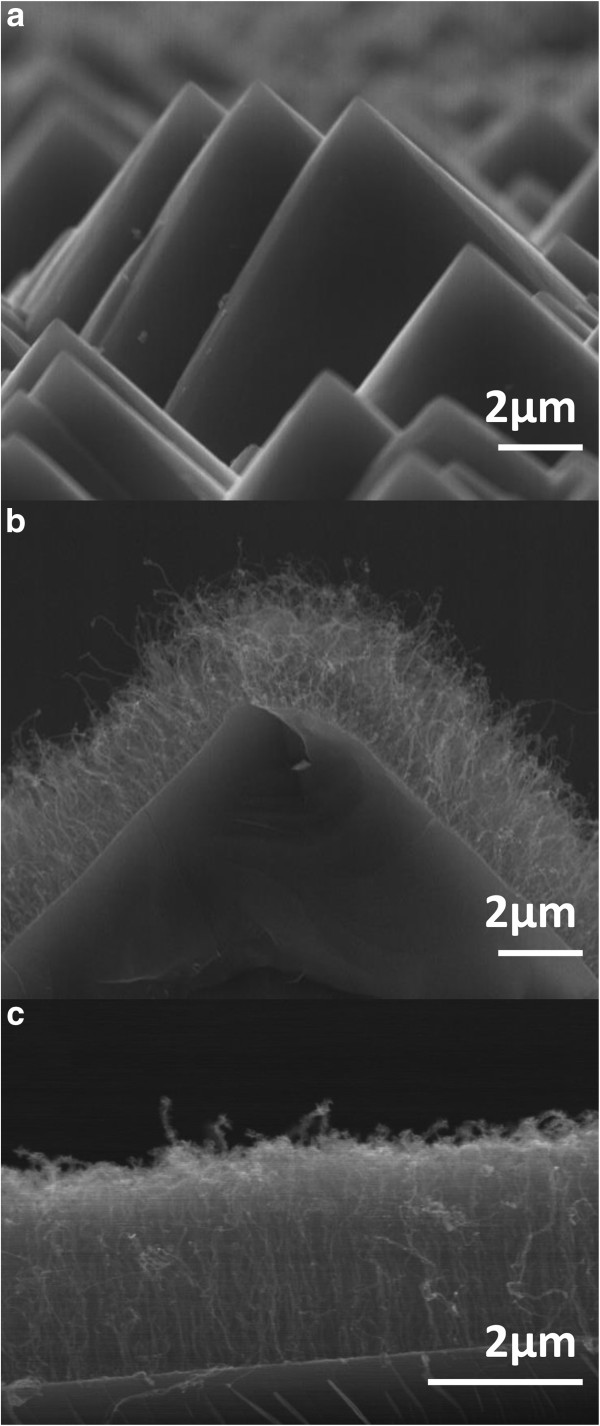
**Typical SEM images. (a)** Pyramidal texturing of the Si (100) substrates after their KOH chemical treatment; **(b)** illustration of the PECVD grown MWCNTs on a silicon pyramid; **(c)** vertically aligned MWCNTs grown by PECVD onto untreated, flat Si (100) substrate.

Figure 1a is a typical scanning electron microscopy (SEM) image of the pyramidal texturing of the Si (100) surface following its KOH chemical treatment. The Si pyramids are generally clean and fairly uniform in size and density. The PECVD growth of the MWCNTs was performed on both pyramidally structured and flat silicon substrates (Figure 1b,c). The MWCNTs were found to always grow perpendicularly to the substrate surface either on the sides of the Si pyramids (as shown by the cross-section SEM view of Figure 1b) or on the untreated flat Si substrates (Figure 1c). This vertical alignment of the MWCNTs with respect to the substrate surface is a consequence of appropriate electrical biasing of the substrate during the plasma growth process (Bower et al. [22]). The growth of MWCNTs was performed under the same PECVD conditions on all the silicon substrates (with various AR values) in order to obtain nearly identical density and morphology of emitters, facilitating thereby their comparison. The SEM images of Figure 1b,c confirm, to a certain extent, the similarity of the MWCNTs whether on Si pyramids or on flat Si substrates. One can nonetheless notice that a minority of MWCNTs protrude from the main nanotube forest (Figure 1b,c). Those protruding emitters, due to their position above the CNT forest canopy, undergo higher electric fields during the FEE measurements.

**Figure 2:**
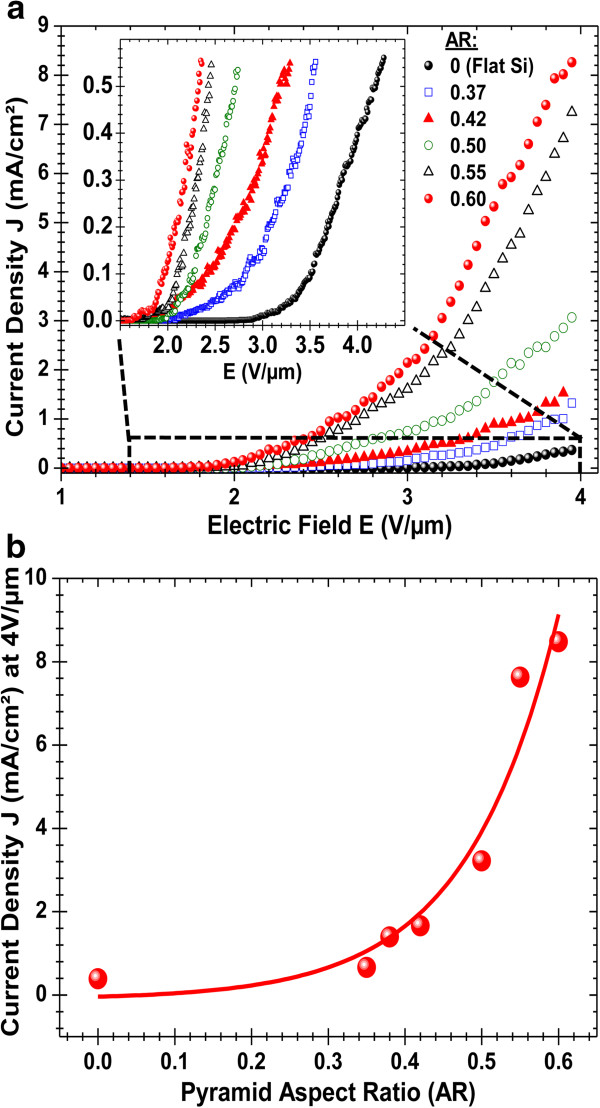
**Field electron emission properties of the developed hierarchal MWCNT cathodes versus their AR. (a)** Typical *J*-*E* curves of the field electron emitting hierarchal MWCNT cathodes with various pyramid AR values along with that of flat Si reference substrate. The inset shows a zoomed-in part of the *J*-*E* curves to compare their threshold field (TF). **(b)** Variation of the emitted current density at an applied field of 4 V/μm as a function of the AR of the Si pyramids.

**Figure 3:**
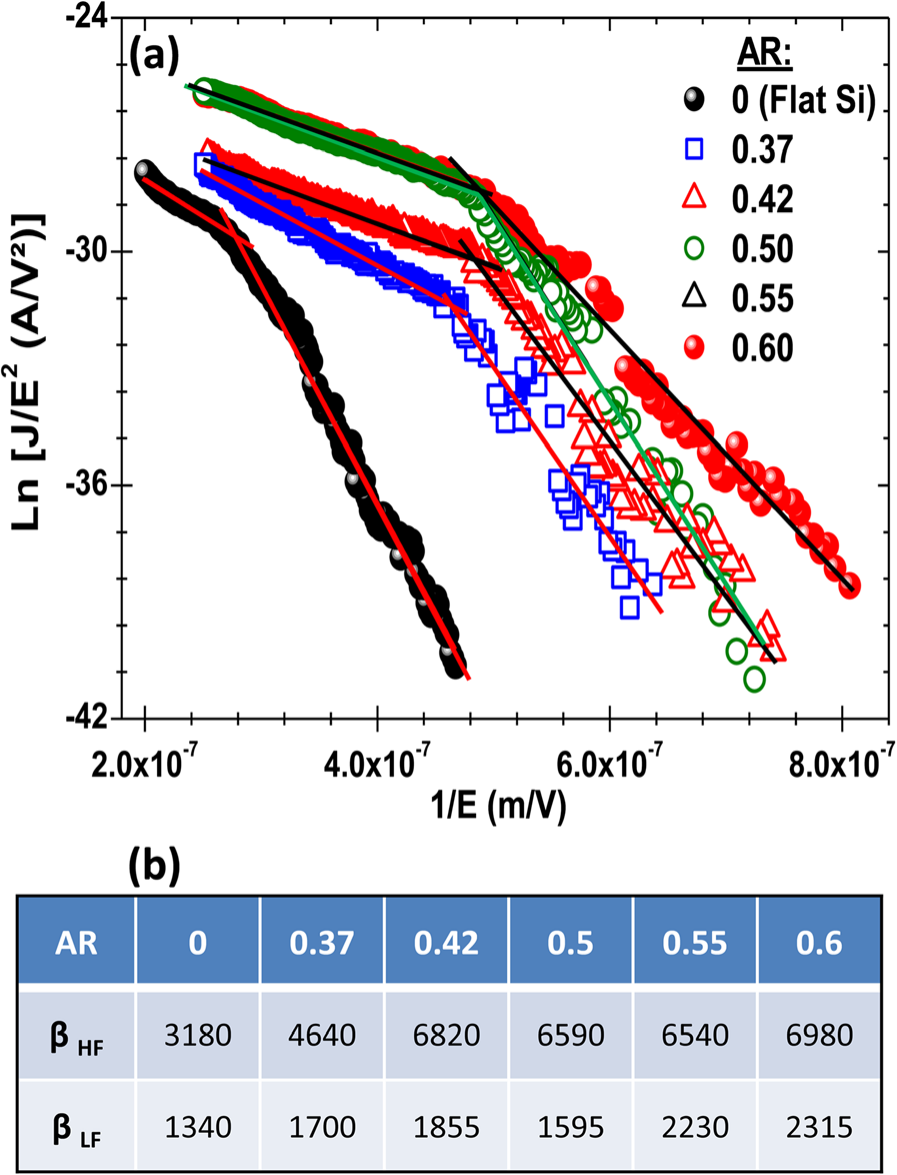
**Fowler-Nordheim analysis of the *J-E *curves of the hierarchal MWCNT cathodes. (a)** Fowler-Nordheim plots for the h-MWCNT cathodes for the various AR values ranging from 0 to 0.6. **(b)** The table summarizes the deduced high-field (HF) and low-field (LF) enhancement factors (*β*) as a function of the AR of the Si pyramids.

Figure 2a shows typical *J*-*E* curves of the developed hierarchal MWCNT cathodes as a function of the AR of the Si pyramids, while comparing them to that of the MWCNTs grown on flat silicon (AR = 0), used here as a non-KOH-treated reference cathode. It is clearly seen that the pyramidal structuring of the cathodes has a significant effect on their FEE performance. Firstly, the inset of Figure 2a shows that as the AR of the Si pyramids is increased, from 0 (flat Si) to 0.6, the *J*-*E* curves are seen to shift progressively towards lower electric field values, indicating a clear decrease of the TF. This TF reduction is thought to be a consequence of the hierarchal structuring of the cathodes as the onset of electron emission occurs at the apex of the pyramids where higher fields are felt by the MWCNTs (Saito & Uemura [3]). Secondly, the *J-E* curves of Figure 2a show that the emitted current density significantly increases as the AR is increased from 0 to 0.6. Indeed, for an electric field of 4 V/μm for example, Figure 2b shows that the current density exponentially increases with the AR. This pyramidal texturing-induced enhancement of the current density is believed to be due to a higher number of MWCNT emitters because of the 3D structuring of the cathodes, which provides larger surface area and lesser screening effect on the pyramid sides. The analysis of the *J*-*E* curves, according to the Fowler-Nordheim (FN) model (Fowler & Nordheim [23]) where the total current density (J in A/m^2^) can be expressed as follows: $J=\frac{A\beta^{2}E^{2}}{\varphi }\;\exp\;\left(\frac{-B\varphi^{\frac{3}{2}}}{\mathrm{\beta E}}\right)$, where *E* is the applied field (V/m), *φ* the work function of the emitter (4.6 eV for MWCNTs (Ago et al. [24]; Su et al. [25])), *A* and *B* are constants with values of 1.56 × 10^−6^ (A·eV/V^2^) and 6.83 × 10^9^ (V·eV^−3/2^ m^−1^), respectively, and *β* is the field enhancement factor that characterizes the ratio between the applied macroscopic field and the local microscopic field felt by the apex of the emitter (Bonard et al. [26]). By fitting the data of Figure 2 to the FN expression, Figure 3 clearly shows that regardless of the AR value of the cathodes, two different domains can be distinguished in the FN plots, namely, high-field (HF) and low-field (LF) regimes. Accordingly, separate *β*_HF_ and *β*_LF_ enhancement factors were extracted from the slopes of the linear fits (Figure 3) and tabulated in the table at the bottom of Figure 3. First of all, in both HF and LF regimes, the enhancement factors are seen to increase significantly (by a factor of 2.2 and 1.7 for *β*_HF_ and *β*_LF_, respectively) as the AR is increased from 0 to 0.6. Respective *β*_HF_ and *β*_LF_ values as high as 6,980 and 2,315 were obtained for the h-MWCNTS cathodes with an AR value of 0.6. This confirms that the hierarchical texturing developed here is effective in enhancing further the local microscopic fields felt by the apex of the MWCNTs. On the other hand, the occurrence of distinct HF and LF regimes in the FN plots of MWCNT emitters has been reported by other groups (Chen et al. [27]; Bai & Kirkici [28]). This indicates that the conventional FN model that describes the FEE of our h-MWCNT cathodes in the LF region cannot be extended to the HF region. Indeed, the evident kink in the FN plots, which is found to occur at the same field value for all the pyramidally texturized cathodes, denotes a clear regime change in the FEE of the MWCNTs. Although there is no consensus about the origin of this regime change (Chen et al. [29]), the enhanced FEE observed in the HF regime is often attributed to space charge effects surrounding the emission sites (Xu et al. [30]; Barbour et al. [31]). Such vacuum space charge buildup is expected to occur more easily on textured substrate with high density of Si pyramids (where higher electric fields are felt by the emitting tips) than on a flat Si cathode (from which some individual nanotubes protrude). This would explain the breakpoint (Figure 3) occurring at rather low-field values in the pyramidally textured cathodes than in the flat Si ones (approximately 2.1 V/μm versus approximately 3.8 V/μm, respectively).

**Figure 4:**
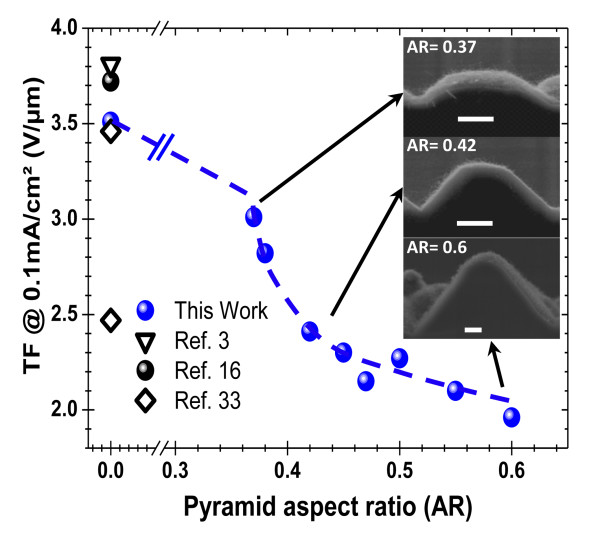
**Threshold field dependence on the aspect ratio of the Si pyramids.** TF values obtained from the flat silicon substrate (AR = 0) from the present work as well as from literature are also included. The inset shows the SEM images of the MWCNT-coated Si pyramids for different AR values (the white scale bar is 2 μm).

To investigate the effect of the AR of the Si pyramids on the TF of the h-MWCNT-based cathodes, while allowing direct comparison with literature, we have defined the TF as the electric field needed to obtain an emitted current density of 0.1 mA/cm^2^. Figure 3 shows that when the AR is varied from 0 (flat Si) to 0.6 (sharp Si pyramids with no mechanical polishing, see the representative SEM images in the inset of Figure 4), the TF significantly decreases from 3.52 to 1.95 V/μm, respectively. This represents a TF value diminution of more than 40% of the initial value of flat Si. It is also worth noting that the latitude of our hierarchal structuring process permits a rather precise tuning of the TF of the h-MWCNT cathodes over all the 1.9 to 3.6-V/μm range. In the case of the flat Si substrates, the measured relatively higher TF value (which compares well with literature data (Futaba et al. [16]; Sato et al. [32]; Wu et al. [33]) as shown in Figure 4) is mainly a consequence of the screening effects between the CNTs (Nilsson et al. [34]). In the flat Si substrate configuration, the highly dense film of vertically aligned CNTs can be approximated to an FEE device consisting of two metal plates facing each other and between which an electric field is applied. In this case, because of the screening effects, the advantage of the high aspect ratio exhibited by the CNTs is not fully exploited, except for the few protruding nanotubes. Using our 3D-textured h-MWCNT cathodes, the electric field lines are concentrated at the tips of the pyramids, resulting into higher fields felt by the CNTs (Saito & Uemura [3]). Moreover, the significant increase of the surface area of the 3D-textured cathodes is also expected to minimize the screening effect between the MWCNTs, particularly on the pyramid sides. Our results clearly demonstrate that the shape of the underlying substrate (i.e., pyramids) has a significant effect on both the TF and current density of the MWCNT cathodes. This corroborates well with the results of the micro-patterned emitters, where the shape of the emitters, more than the pitch between them, was reported to play a more important role in the FEE properties of the CNT cathodes (Sato et al. [32]).

## Conclusions

We have developed a relatively straightforward, effective, and affordable approach to achieve hierarchal 3D structuring of the h-MWCNT-based cold cathodes. Our process is based on the optimized PECVD growth of MWCNTs onto pyramidally KOH-texturized silicon (100) substrates. By varying the aspect ratio of the Si pyramids, we were able to show the significant improvement of the FEE properties of the h-MWCNT cathodes, compared to their Si flat counterparts. In particular, our results show that the higher the AR of the Si pyramids, the lower the TF of the h-MWCNT cathodes. A TF value as low as 1.95 V/μm was achieved for the h-MWCNT cathodes with an AR value of 0.6 (a decrease of more than 40%, compared to MWCNT forest grown on flat Si substrates). The effectiveness of our approach is also reflected by the higher enhancement factors in both low- and high-field regimes. The prospect of a relatively easy scale up of the hierarchal structuring process developed here makes this approach highly attractive for applications where low-cost and large-surface cold cathodes are needed.

## Competing interests

The authors declare that they have no competing interests.

## Authors’ contributions

LAG performed most of the experimental work including the PECVD synthesis of the MWCNTs and FEE characterizations of the cold cathodes. VLB contributed to the characterizations work (particularly the SEM observations) and to the analysis of the FEE data. SA provided general feedback on the progress of the project and corrections to the manuscript. MAE supervised the entire process and suggested experiments while providing critical feedback all along the progress of the project. He also corrected the manuscript and finalized its drafting. All authors read and approved the final manuscript.

## Authors’ information

LAG is currently a Ph.D. student at the Institut National de la Recherche Scientifique. His Ph.D. project focuses on the PECVD synthesis of carbon nanotubes and the study of their field-emission properties under different novel architectures (such as the *hierarchal* cathode-based devices reported here). He authored and/or co-authored four scientific papers so far. VLB is currently a postdoctoral researcher at the Institut National de la Recherche Scientifique, where he works on laser-based synthesis of various nanomaterials (including carbon nanotubes and quantum dots), their optoelectronic characterizations, and integration into devices. He has particularly developed single-wall carbon nanotubes and silicon hybrid solar cells. His research contributions include 12 published papers in prestigious journals and participation to more than 15 national and international conferences. SA is the president of pDevices, Inc. He received his Ph.D. in Experimental Atomic and Ionic Physics from the University of Paris-Sud (Paris XI). He has more than 20 years of experience in atomic and ionic physics-based instrumentation as well as in the management of industrial projects. He developed various spectrometry instruments while working at different prestigious light source labs in France, Germany, USA, and Canada. He is currently developing at pDevices innovative technologies for automatic, real-time early detection, and diagnosis and prevention of adverse health conditions. MAE is a Full Professor and the leader of the ‘NanoMat’ Group, he founded in 1998 at the Institut National de la Recherche Scientifique (INRS-EMT, Varennes, Quebec, Canada). He is an internationally recognized expert in the inter-related fields of laser/plasma-based synthesis, modification, nanoassembly, and characterizations of nanostructured materials (including nanotubes, nanoparticles, nanohybrids, and thin films) and their applications for advanced devices. He has published more than 160 refereed publications in prestigious journals, and he is a co-inventor of five patents. His research contributions are well cited (his current H index is 25). The R&D contributions and expertise of MAE are well recognized at both national and international levels, as testified by his numerous invited talks, appointments as a scientific reviewer for various public and private R&D funding agencies, as a board member of steering committees of R&D Canadian organizations, and as a member of international scientific advisory boards and/or session chair at international conferences. He is currently a member of the editorial board of the ISRN-Nanotechnology and Scientific Reports (from the Nature Publishing Group) journals. He is also a regular reviewer of more than 20 journals in the fields of materials, nanoscience, and nanotechnology.
